# Spatiotemporal profiling of adhesion G protein-coupled receptors in developing mouse and human pancreas reveals a role for GPR56 in islet development

**DOI:** 10.1007/s00018-025-05659-z

**Published:** 2025-03-26

**Authors:** Oladapo E. Olaniru, Klaudia Toczyska, Nunzio Guccio, Stefanie Giera, Xianhua Piao, Aileen J. F. King, Peter M. Jones, Shanta J. Persaud

**Affiliations:** 1 Department of Diabetes, School of Cardiovascular and Metabolic Medicine & Sciences, King’s College London, Guy’s Campus, London, SE1 1UL UK; 2 Department of Medicine, Children’s Hospital, Harvard Medical School, Boston, MA 02115 USA; 3 Department of Pediatrics, University of California at San Francisco, San Francisco, CA USA

**Keywords:** Pancreatic endocrine progenitors, Adhesion GPCRs, GPR56, Pancreas development, ScRNAseq

## Abstract

**Introduction:**

G protein-coupled receptors (GPCRs) are cell-surface proteins that are targeted therapeutically for a range of disorders, including diabetes. Adhesion GPCRs (aGPCRs) are the second largest class of the GPCR superfamily and some members of this family have been implicated in appropriate organ development. However, the role of aGPCRs in endocrine pancreas specification is not yet known.

**Methods:**

Here, we systematically characterised expression of mRNAs encoding aGPCRs and their ligands in developing mouse and human pancreas using our own and publicly available single-cell RNA sequencing and spatial transcriptomics data, and we conducted qPCR analysis of aGPCR expression in human pancreas at different gestational stages. We then investigated the role of GPR56 (ADGRG1), the most abundant aGPCR in pancreatic endocrine progenitors, in islet development using *Gpr56* null mice and their wildtype littermates.

**Results:**

We demonstrated that aGPCRs are dynamically expressed during mouse and human pancreas development, with specific aGPCR mRNAs expressed in distinct endocrine, endothelial, mesenchymal, acinar, ductal, and immune cell clusters. aGPCR ligand mRNAs were mostly expressed by non-endocrine cells, and the most highly expressed receptor-ligand interacting mRNA pairs were those encoding GPR56 and COL3A1. Deletion of *Gpr56* in neonatal mice was associated with an altered α-/β-/δ-cell ratio and reduced β-cell proliferation.

**Conclusion:**

Our data show that aGPCRs are expressed at key stages of human and mouse pancreas endocrine lineage decisions, and analysis of pancreases from *Gpr56* knockout mice implicate this aGPCR in the development of a full complement of β-cells.

**Supplementary Information:**

The online version contains supplementary material available at 10.1007/s00018-025-05659-z.

## Introduction

Islets of Langerhans are pancreatic endocrine micro-organs that play a major role in maintaining glucose homeostasis, dysregulation of which results in diabetes. The specification and maturation of pancreatic progenitor cells into terminally differentiated islet cells is largely governed by transcription factors, in particular those belonging to the helix-loop family such as Ngn3, Pdx1 and Pax4 [1–4]. Recent studies have shown that cell-membrane anchored proteins such as those involved in extracellular matrix interactions may play crucial roles in islet ontogeny [5–8]. Adhesion G protein-coupled receptors (aGPCRs) are a family of 33 atypical GPCRs that have several unique characteristics, including the presence of a long, multi-motif N-terminal domain and a conserved domain, and they undergo self-cleavage, splitting the receptor into non-covalently attached N- and C-terminal fragments [9]. aGPCRs mediate cell-cell and cell-extracellular matrix interactions and they are important for the appropriate development of several cells and organs including glia, oligodendrocytes, cerebral cortex, kidney and lungs [10–17]. A recent report has implicated GPR116 (ADGRF5) in islet development, with observations of reduced β-cell mass following global Gpr116 deletion in mice [18]. However, the overall role of aGPCR family members in islet development is not yet clear, and understanding their importance is made more difficult by the majority of the members of this GPCR sub-family being orphans.

There have been several reports on roles for the aGPCR GPR56 (ADGRG1) in cell development and function. Thus, it is important for the proper formation of the cerebellum, as it allows the developing neurons to adhere to the extracellular matrix of the pial basement membrane, failure of which leads to neuronal over-migration and ectopia [19, 20]. Moreover, loss of Gpr56 in mice leads to hypomyelination of the CNS as a result of defective development of oligodendrocyte precursor cells [10]. In humans, a loss of function mutation in GPR56 leads to an inherited brain disorder known as bilateral frontoparietal polymicrogyria, where the patients present with moderate to severe mental retardation, seizures, poor eye-muscle coordination and cerebral hypoplasia [15, 21, 22]. In addition, Gpr56 plays structural functions in seminiferous tubule development in mice, where its absence during embryogenesis leads to reduced male fertility [23]. We, and others, have reported that it is the most abundant islet-expressed GPCR in adult human and mouse islets, where it is activated by the extracellular matrix protein collagen III [24–27]. Gpr56 activation by collagen III in mouse islets is associated with potentiation of glucose-induced insulin secretion and reduced β-cell apoptosis, but Gpr56 is not required for mouse islet vascularisation or innervation, and its deletion does not impair glucose tolerance in adult mice [27]. While there have been no investigations on the role of GPR56 in islet development it is highly enriched in Ngn3-positive mouse pancreas endocrine progenitors [28], suggesting that it could be of importance in mediating endocrine cell differentiation.

In the current study we used our own and publicly available single-cell RNA sequencing (scRNAseq) and spatial transcriptomic datasets, together with qPCR, to characterise expression patterns and spatial localisation of mRNAs encoding aGPCRs and their ligands within developing mouse and human pancreases. We also investigated the role played by Gpr56, one of the most abundant aGPCRs in the developing pancreas, in islet development and maturation using *Gpr56* knockout (*Gpr56* KO) mice.

## Materials and methods

### Materials

All general laboratory reagents and cell culture materials used in this study were obtained from Sigma-Aldrich (Dorset, UK) unless otherwise stated. RNeasy mini kits, QuantiTect SYBR Green qPCR kits and primers were purchased from Qiagen (Manchester, UK) and the RNAscope 2.5 HD Red Detection Kit, Target Retrieval solution and GPR56 mRNA probe were from Advanced Cell Diagnostics (Oxford, UK).

### Data mining of mouse and human pancreas single cell RNAseq and spatial transcriptomics data

Integrated scRNAseq data of 4,620 cells from mouse pancreas at embryonic days E12.5, E13.5, E14.5, E15.5 and E18.5 [29] were downloaded from https://cells.ucsc.edu/?bp=pancreas and processed in Seurat [30]. Briefly, the Seurat object was first updated to version 3.0 and processed for dot plot visualisation. In addition, our scRNAseq data containing 53,204 cells of the developing human pancreas at post-conception weeks (PCW) 12, 13, 14, 15, 18, 19 and 20 and Visium spatial transcriptomics data at 15 PCW [31] were also processed using standard Seurat workflow and receptor-ligand hotspots in spatial transcriptomics were visualised by stLearn [32], as we have described previously [31].

### Human fetal pancreases

Developing human pancreases at post-conception weeks (PCW) 8–20 were obtained from the MRC-Wellcome Trust Human Development Biology Resource from terminations of pregnancy with appropriate ethical approval from London-Fulham and Newcastle & North Tyneside 1 Research Ethics Committees. Samples were either frozen immediately upon receipt in RNAlater at -80^°^C until RNA extraction or fixed in 4% paraformaldehyde for immunohistochemical analysis.

### Human fetal pancreas RNA extraction and quantitative real-time PCR

Human fetal pancreases were homogenised at 4^°^C in lysis buffer, total RNAs were extracted using an RNeasy mini kit according to the manufacturer’s protocol and RNAs were then reverse transcribed to cDNAs. Quantitative PCR using SYBR green primers was performed to determine the expression of aGPCRs and five reference genes (GAPDH, ACTB, PPIA, TBP and TRFC) at different stages of human pancreas development [33]. The human fetal pancreas qPCR data were normalised relative to the average of the three most stable reference genes identified by RefFinder [34].

### Mice

*Gpr56* KO mice and their wildtype (WT) littermates were maintained as colonies at Children’s Hospital Boston, USA, and ethical approval was obtained from the Animal Use and Care Committee of Boston Children’s Hospital. For analysis of islet endocrine cell proliferation *Gpr56* KO and WT mice at postnatal day 9 (P9) were injected intraperitoneally once with 50 mg/kg BrdU and pancreases were retrieved 24 h later.

### Immunohistochemistry

Freshly retrieved mice and human fetal pancreases were fixed in 4% paraformaldehyde and processed with an automated tissue processor (Leica Biosystems) before being wax-embedded. 5 μm thick sections were de-waxed, rehydrated and antigenicity was restored by boiling in 10mM sodium citrate buffer, pH 6. Sections were incubated in blocking buffer (1% BSA, 10% normal goat serum, 0.1% triton-X-100 in PBS) for 1 h at room temperature. The following primary antibodies were added to the sections and incubated overnight at 4^°^C: rabbit anti-GPR56 (generated by Yenzym Antibodies, 1:250) [20], guinea-pig anti-insulin (Dako, 1:500), mouse anti-glucagon (Sigma-Aldrich, 1:50), rat anti-somatostatin (Invitrogen, 1:25), mouse anti-BrdU (Merck Life Science, 1:100) and rat anti-BrdU (AbD Serotec, 1:100). After three washes in PBS, pancreas sections were incubated with Alexa-fluor 488- or 594-conjugated secondary antibodies (Jackson ImmunoResearch, 1:100) for 1 h followed by nuclei counterstaining with DAPI (1:2000). Images were captured using a confocal microscope (Nikon Eclipse Ti-E inverted) or fluorescence microscope (Nikon TE 2000-U inverted).

### In situ hybridisation by RNAScope 2.5 HD

Human fetal pancreas blocks were sectioned, de-paraffinised and antigen retrieval was carried out by boiling slides in Target Retrieval solution at 98-102^°^C for 15 min. Sections were dehydrated in ethanol with subsequent protease treatment before incubation with a GPR56 probe at 40^°^C for 2 h. A probe targeting the human housekeeping gene peptidyl-propyl isomerase B (PPIB) served as a positive control, while a probe targeting the bacterial DapB gene acted as a negative control. Nuclei were counterstained with Gill’s haematoxylin and 0.02% ammonium hydroxide and signals were developed with a Fast Red solution, according to the manufacturer’s protocol. The number of mRNA dots per cell was quantified by ImageJ using the Weka classifier plugin for segmentation.

### Statistical analyses

Statistical analyses were performed using Graph Prism 8.0. Statistical significance was obtained using two-tailed Student’s t-tests or ANOVA, as appropriate, with Bonferroni’s post hoc tests. For histological analyses images were scored blindly before quantification.

## Results

### Expression of adhesion GPCRs in developing mouse and human pancreatic cells

**Fig. 1:**
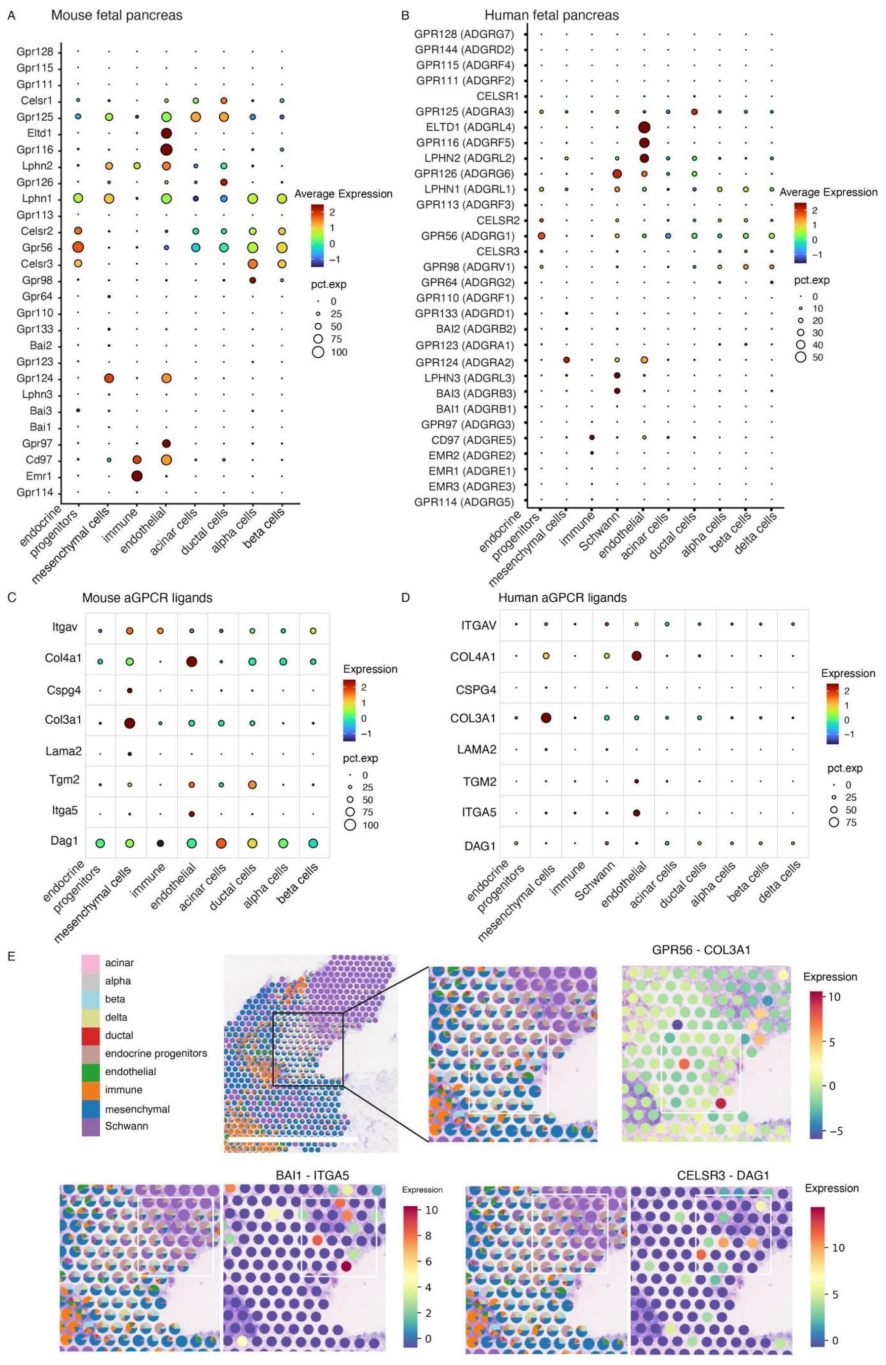
Single cell RNA sequencing reveals adhesion GPCR mRNA expression patterns in developing mouse and human pancreas. **A**) A dot plot showing expression of aGPCR mRNAs in different cell types of the developing mouse pancreas. The size of the dot represents the proportion of cells expressing the gene of interest, and colour indicates expression levels. The data are from scRNAseq analysis of mouse embryonic pancreases from E12 to E18, containing 4,620 cells [29]. Data for Schwann cells and delta cells are not available in the mouse pancreas dataset. **B**) A dotplot showing expression of aGPCR mRNAs in different cell types of the developing human pancreas. The data are from our scRNAseq analysis of human fetal pancreases from 8 PCW to 20 PCW, containing 53,204 cells [31]. **C**) A dot plot showing expression of aGPCR ligand mRNAs in mouse embryonic pancreas [29]. **D**) A dot plot showing expression of aGPCR ligand mRNAs in developing human pancreas from our data [31]. **E**) A Visium human pancreas spatial transcriptomics image, with cell type prediction at 15 PCW (Left panel). Spatial distribution of receptor-ligand gene expression for GPR56-COL3A1, BAI1-ITGA5 and CELSR3-DAG1 are shown in the three labelled panels. The boxed area indicates the region containing endocrine progenitors and mesenchymal, Schwann and endothelial cells, which produce the majority of the ligands that may interact with aGPCRs on the endocrine progenitors. Scale bar: 2 mm

We analysed published scRNAseq data from developing mouse pancreases (embryonic stages E12 to E18) [29] and our data from human fetal pancreases (8 to 20 PCW) [31] to identify multiple aGPCRs in mouse and human pancreases, with varying levels of expression in different pancreatic cell types (Fig. 1A and B). We confirmed expression of canonical gene markers in both mouse and human pancreatic cell types, as shown in Supplementary Fig. 1. GPR112 was not detected in the human scRNAseq data nor was EMR4, which is a pseudogene [9]. It was observed that in both mouse and human pancreases there was conserved expression of LPHN1, CELSR2, GPR56, and CELSR3 in endocrine progenitors and β-cells, CD97 in immune cells, ELTD1, GPR116, GPR124 and LPHN2 in endothelial cells, and GPR124 in mesenchymal cells. The immune cell aGPCR repertoire in human fetal pancreas included low levels of CD97 and EMR2 while Cd97, Emr1 and Lphn2 mRNAs were readily detected in embryonic mouse pancreas immune cell populations. Given the importance of endocrine progenitor cells in β-cell specification, the observation that GPR56 was the most highly expressed aGPCR mRNA in both mouse and human endocrine progenitors (Fig. 1A and B) suggests a role for GPR56 in β-cell development.

To investigate whether the aGPCRs expressed in developing human pancreas were also expressed in adult pancreas we interrogated the Tabula Sapiens scRNAseq data of 15 pancreases from normal subjects [35]. Focusing on the endocrine cell types, we identified that GPR98 mRNA, which was expressed by fetal α-, β- and δ-cells (Fig. 1B) was confined to α- and PP-cells in adult endocrine pancreas (Suppl. Figure 2). The data in adult human pancreas also indicated that mRNAs encoding GPR56 and LPHN1, two aGPCRs that were detected in fetal human β-cells (Fig. 1B), were most highly expressed in β-cells, suggesting that GPR56 and LPHN1 may be important for both β-cell development and function.

We next investigated expression of aGPCR ligand mRNAs in developing mouse and human pancreas cell populations to determine whether aGPCR ligand interactions with their cognate receptors could be functionally relevant in pancreas development. We focused on aGPCR endogenous ligands identified so far, which are mainly components of the extracellular matrix [36], although the activation mechanism of some ligands is not fully clear and some are referred to as interacting partners. Transcripts encoding ligands/interacting partners such as integrin-αVβ3 (ITGAV), collagen IV (COL4A1), collagen III (COL3A1), laminin-211 (LAMA2), transglutaminase 2 (TMG2) and dystroglycan (DAG1) were identified in both developing mouse and human pancreases and they were expressed predominantly by mesenchymal and endothelial cells in both species (Fig. 1C and D). COL3A1 and TMG2 are GPR56 ligands [19, 27, 37], COL4A1 and LAMA2 are GPR126 ligands [38, 39], while DAG1 and ITGA5 interact with CELSR3 [40] and BAI1 [41] respectively. Our spatial transcriptomics analysis, using Visium gene expression slides with a resolution per spot of 55 μm, indicated that in human fetal pancreas at 15 PCW there was a spatial region comprised of endocrine progenitors, Schwann, mesenchymal and endothelial cells that showed high co-expression of GPR56-COL3A1, BAI1-ITGA5 and CELSR3-DAG1 (Fig. 1E). This high co-expression of aGPCRs and their interacting partners suggests sites of aGPCR-ligand interactions within these cell populations and that GPR56, BAI1 and CELSR3 expression may be functionally relevant during the development of pancreatic endocrine progenitors by interacting with ligands present within the extracellular matrix micro-environment. However, the spatial transcriptomics observations do not definitively indicate that these interactions occur in the developing pancreas, and further experiments using specific antibodies directed against the aGPCRs and interacting partners are required to determine whether this is the case.

### Temporal profile of adhesion GPCR mRNA expression in developing human pancreas

To investigate the temporal pattern of aGPCR mRNA expression in developing human pancreas, we carried out quantitative PCR analysis using fetal samples at multiple timepoints that represent important developmental milestones: Carnegie stages 19 to 23 (CS19-23), where the first NGN3 + and insulin + cells are detectable, indicative of endocrine commitment [42, 43]; 10 PCW and 12 PCW, which are characterised by acini differentiation, initiation of islet vascularisation and islet clustering [44, 45]; 17 PCW and 19–21 PCW, in which there is expansion of pancreatic epithelium and islet clusters [46].

We first ascertained the reliability of our qPCR profiling by assessing variability in expression of five reference genes (GAPDH, ACTB, PPIA, TBP and TRFC) that we have previously found to be stably expressed in adult mouse and human islets [33]. In developing human pancreas, GAPDH, ACTB and PPIA were highly expressed across the different developmental stages, with average Ct values ranging from 13.5 to 16.6, while TBP and TRFC showed lower expression (average Ct values of 19.1 to 21.8) (Suppl. Figure 3A). The stability of the reference genes across the developmental timepoints was evaluated using RefFinder [34], a comprehensive algorithm that ranks reference gene stability using computational programmes such as BestKeeper [47], Normfinder [48], geNorm [49] and Delta-Ct [50]. The comparative ΔCt method ranked PPIA, TBP and ACTB as the most stable genes while GAPDH and TRFC were the least stable (average STDEV: PPIA: 0.50, TBP: 0.54, ACTB: 0.54, GAPDH: 0.76, TRFC: 0.78) and the four programs consistently ranked PPIA and TRFC as the most stable and the least stable genes, respectively (Suppl. Figure 3B). We therefore normalised aGPCR expression in human fetal pancreases to the three most stable genes: PPIA, TBP and ACTB.

**Fig. 2:**
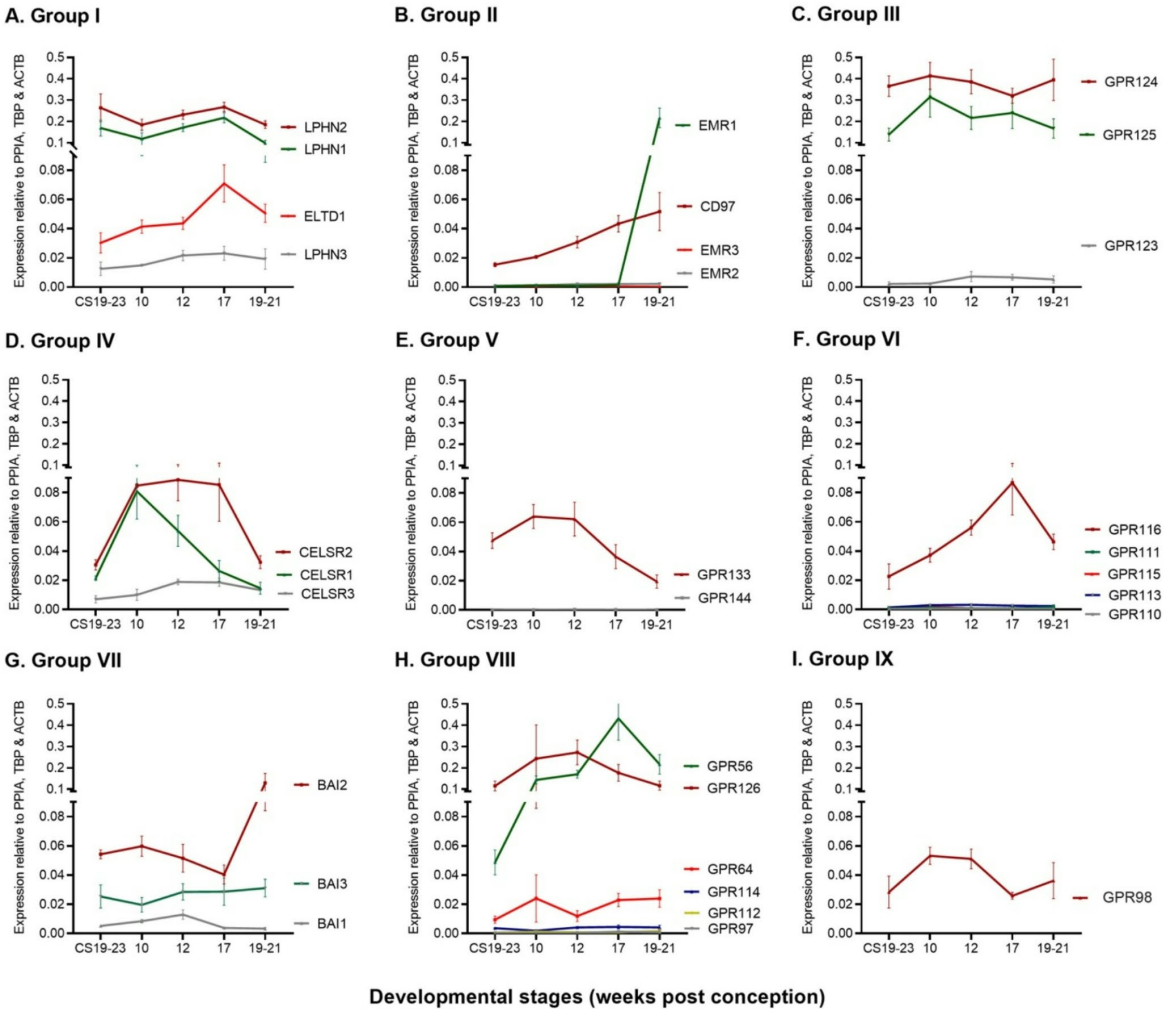
Expression profile of adhesion GPCR mRNAs in developing human pancreas. **A**)– **I**) RT-qPCR quantification of adhesion GPCR mRNA expression relative to three reference genes, PPIA, TBP and ACTB, in developing human pancreases retrieved from Carnegie stages 19–23 (CS19-23) to 21 weeks post conception. Adhesion GPCR gene expression has been classed according to their sub-groups, Group I to Group IX, at multiple timepoints of human pancreas development. Data are presented as mean±SEM, *n* = 3–4 pancreases at each time point

9 distinct subclasses of aGPCRs have been defined, based on their N-terminal extracellular functional domains [9]. It can be seen from Fig. 2 that different patterns of aGPCR mRNA expression were observed within individual subclasses during human fetal pancreas development, perhaps reflecting the variability in N-terminal domain structure, even within subclasses. LPHN1, LPHN2, GPR124, GPR125, GPR56, and GPR126 were the most abundant aGPCR mRNAs during development while there was very low expression of mRNAs encoding EMR2, EMR3, GPR144, GPR113, GPR115, GPR111, GPR110, GPR114 GPR112 and GPR97 (Fig. 2). GPR124, an essential gene for development of the CNS vasculature [51, 52], was the most highly expressed aGPCR at the five timepoints except at 17 PCW where GPR56 expression was higher (Fig. 2C and H). EMR1 mRNA was barely detectable at the early stages of human pancreas development but at 19–21 PCW there was a 400-fold increase (*p* < 0.001), making it the most upregulated aGPCR (Fig. 2B). GPR56 was the second most upregulated aGPCR mRNA, with ∼ 10-fold increase (*p* < 0.01) in expression between CS 19–23 and 17 PCW (Fig. 2H). ELTD1, CD97, GPR116 and GPR56 mRNAs showed steady upregulation until 17 PCW (*p* < 0.05), while GPR133 and CELSR1 were significantly downregulated between 10 PCW and 19–21 PCW (*p* < 0.05 and *p* < 0.01 respectively). However, there were no significant changes in the expression of mRNAs encoding LPHN1, LPHN2, LPHN3, EMR2, EMR3, GPR123, GPR124, GPR125, GPR144, GPR110, GPR111, GPR113, GPR115, BAI1, BAI3, GPR64, GPR97, GPR112, GPR114, GPR126 and GPR98 during human pancreas development.

### Expression of GPR56 in developing mouse and human pancreas

**Fig. 3:**
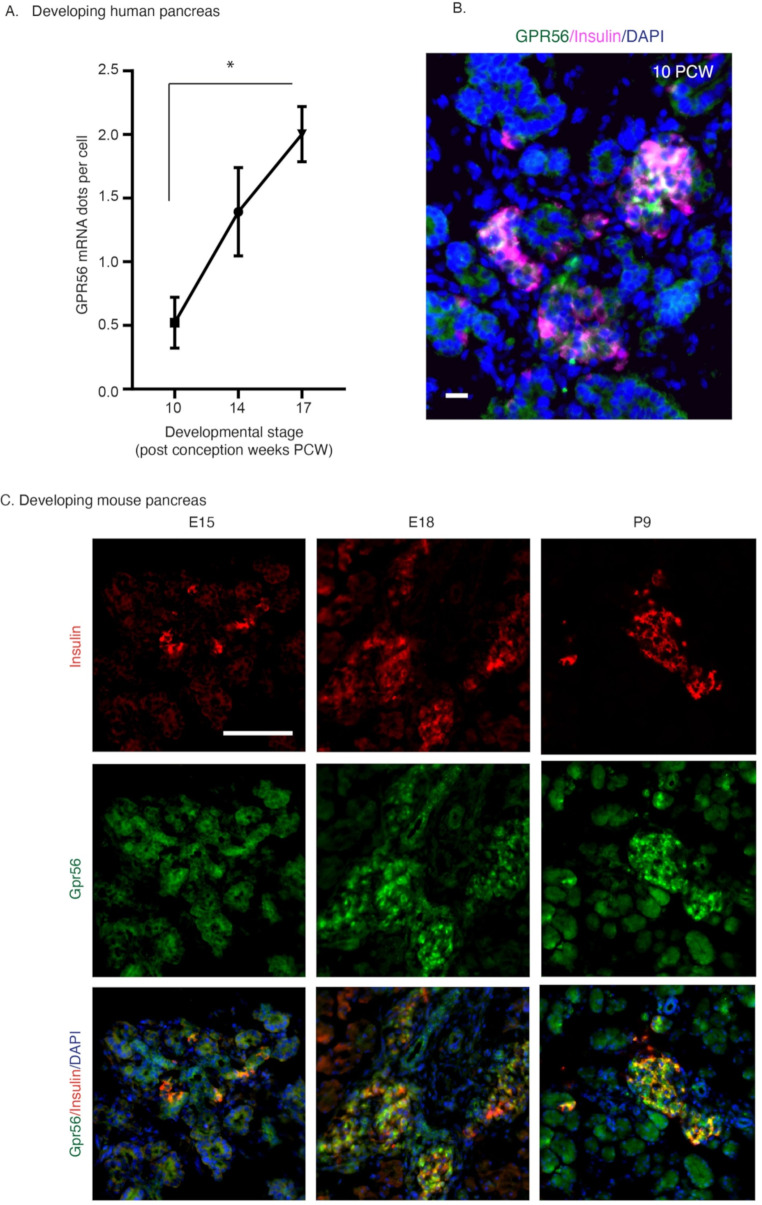
GPR56 is highly expressed in developing human and mouse pancreases. **A**) Quantification of GPR56 mRNA dots per cell in human fetal pancreases at 10, 14 and 17 PCW. Data are mean±SEM, *n* = 3 human pancreases at each time point, *p < 0.05, one-way ANOVA with Bonferroni’s post-hoc test. **B**) Representative immunohistochemical image showing GPR56 expression in developing human pancreas at 10 PCW. GPR56 (green) is expressed by differentiated islet clusters at this stage of gestation, where it mainly co-localises with insulin immunoreactivity (magenta). Scale bar: 20 μm. **C**) Representative immunohistochemical panels showing Gpr56 expression (green) and its colocalisation with insulin (red) in developing mouse pancreases at embryonic day 15 (E15), E18 and postnatal day 9 (P9). Scale bar: 50 μm

To further confirm that GPR56 is progressively upregulated as human islets develop we carried out orthogonal validation of GPR56 mRNA upregulation in developing human pancreases using RNAscope 2.5 HD in situ hybridisation. We first validated the technique in human fetal pancreas using a human PPIB positive control probe and a negative control probe targeting the bacterial DapB gene (Suppl. Figure 4A), and also the GPR56 gene at CS13 (Suppl. Figure 4B). The RNAscope assay indicated that GPR56 mRNA transcripts, counted as mRNA dots per cell by ImageJ, showed a steady, significant increase in pancreases between 10 and 17 PCW (Fig. 3A; Suppl. Figure 4C-E). Fluorescence immunostaining of human pancreas showed that GPR56 immunoreactivity had a high degree of co-localisation with insulin as early as 10 PCW (Fig. 3B), and Gpr56 expression by beta cells was also evident in mouse fetal (E15, E18) and postnatal (P9) pancreases (Fig. 3C). In mouse pancreas at E15, Gpr56 was expressed by almost all cell types but it gradually became restricted to the islet cells, with increased co-localisation of Gpr56 and insulin staining at E18 and P9 (Fig. 3C), suggesting that Gpr56 may be required as the β-cells develop and form clusters.

### Effect of GPR56 deletion on mouse islet development

**Fig. 4:**
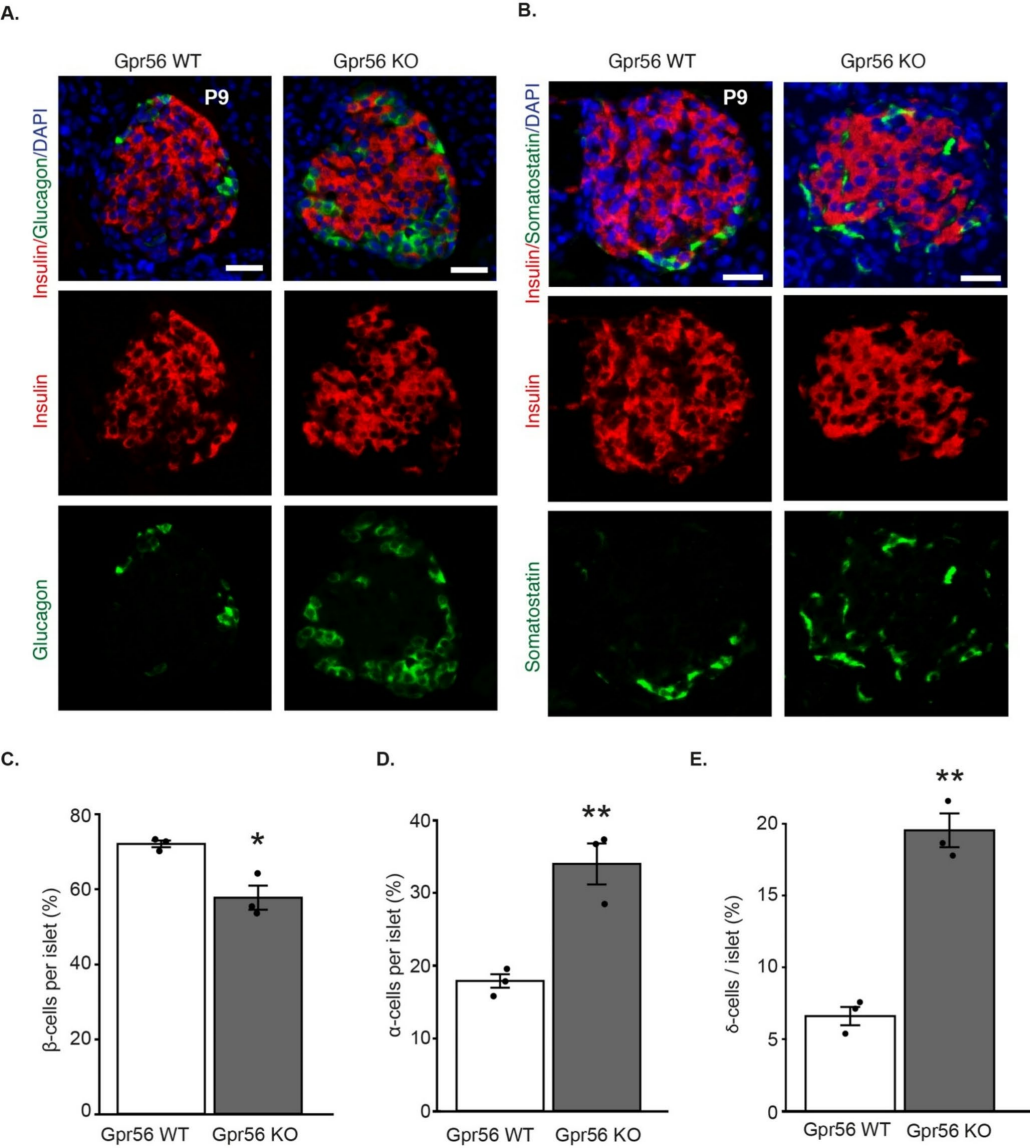
Global deletion of *Gpr56* leads to altered α-/β-/δ-cell ratios in postnatal mice. **A**) Representative immunohistochemical images showing expression of insulin (red) and glucagon (green) in wildtype (WT) and *Gpr56* KO mouse pancreases at P9. DAPI nuclear stain is shown in blue. Scale bar: 50 μm. **B**) Representative immunohistochemical images showing expression of insulin (red) and somatostatin (green) in WT and *Gpr56* KO mouse pancreases at P9. DAPI nuclear stain is shown in blue. Scale bar: 25 μm. **C**-**E**) Percentage of insulin-positive β-cells (**C**), glucagon-positive α-cells (**D**) and somatostatin-positive δ-cells **E**) per islet in WT (open bars) and *Gpr56* KO (closed bars) mouse pancreases at P9. Data are mean ± SEM of 8 islets each from 3 mice per genotype, *p < 0.05, unpaired t-test; **p < 0.01, unpaired t-test

**Fig. 5:**
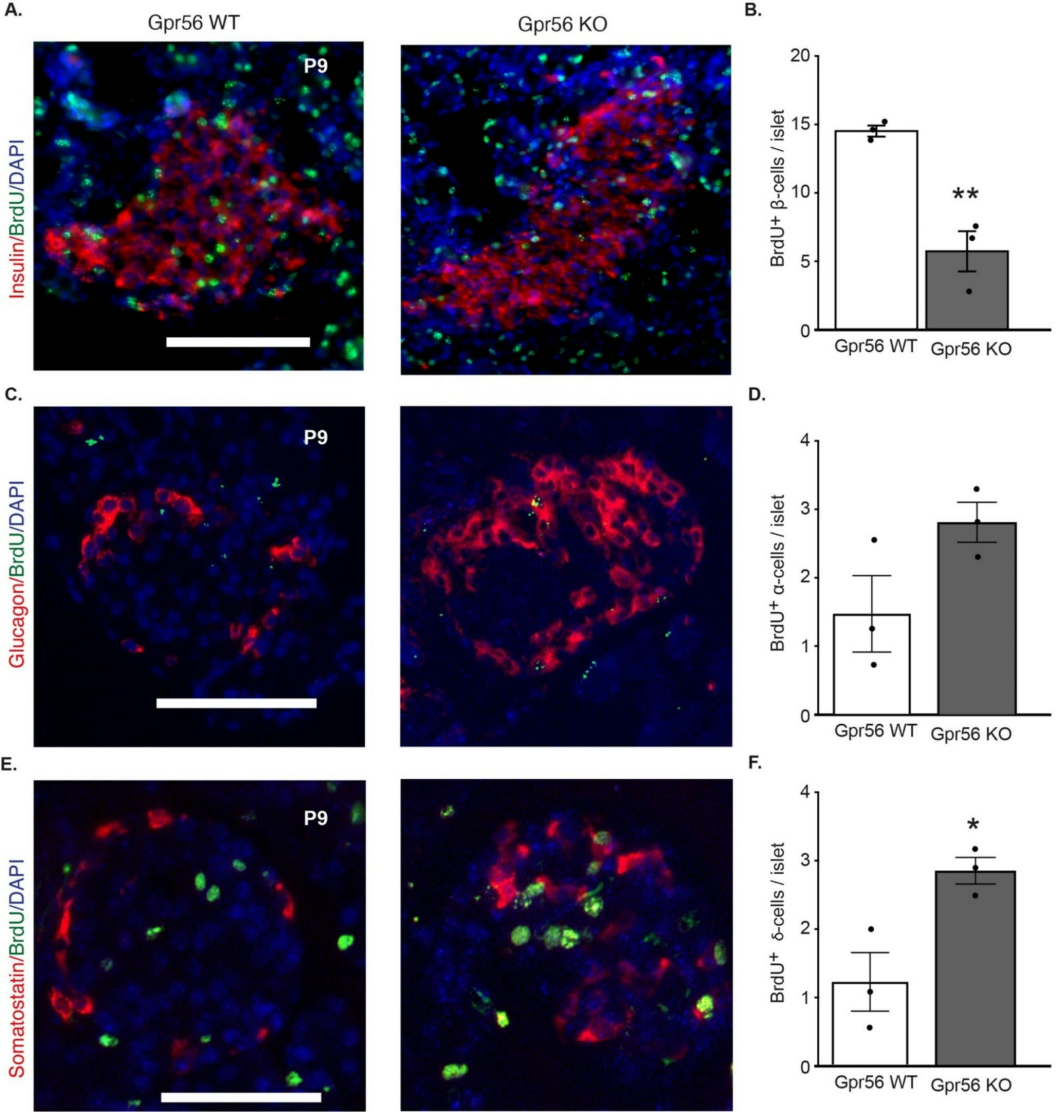
Global deletion of *Gpr56* leads to altered islet endocrine cell proliferation in postnatal mice. **A**, **C**, **E**) Representative immunohistochemical images showing expression of insulin (red) and BrdU (green; **A**), glucagon (green) and BrdU (red; **B**) and somatostatin (red) and BrdU (green; **C**), in WT (left panels) and *Gpr56* KO (right panels) mouse pancreases at P9. DAPI nuclear stain is shown in blue. Scale bar: 50 μm. **B**, **D**, **F**) Double positive cells for BrdU and insulin (**B**), glucagon (**D**) and somatostatin (**F**) were quantified in pancreases retrieved from P9 WT (open bars) and *Gpr56* KO (closed bars) mice that were culled 24 h after intraperitoneal BrdU administration. Data are mean±SEM of 4 islets each from *n* = 3 mice per genotype, *p < 0.05, unpaired t-test; **p < 0.01, unpaired t-test

These observations in developing human and mouse pancreas suggest a role for GPR56 in β-cell development. Next, we investigated whether *Gpr56* deletion had an impact on islet endocrine cell distribution, using a global *Gpr56* knockout mouse model that we have previously characterised [27]. The numbers of β-, α-cells and δ-cells at P9, a period associated with a rapid increase in β-cell proliferation [53], were determined by immunostaining for insulin, glucagon and somatostatin in pancreases from wildtype and *Gpr56* KO mice. It can be seen from Fig. 4A and B that islets at P9 contained cells expressing the three major islet hormones, insulin, glucagon and somatostatin, and Gpr56 deletion did not affect islet morphology. The number of islet cells per section was not significantly altered in pancreases from *Gpr56* KO mice (WT: 90.4±5.1; KO: 76.5±7.3, *n* = 3 mice per genotype, *p* = 0.19) but analysis of multiple P9 pancreatic sections indicated that there was a significant decrease in the proportion of β-cells in pancreases of *Gpr56* KO mice (Fig. 4C). This reduction in β-cells was accompanied by a significant increase in α-cells and δ-cells (Fig. 4D and E) following deletion of *Gpr56*. Immunoprobing for BrdU^+^ β-cells in pancreases from P9 mice revealed a significant reduction following *Gpr56* deletion (Fig. 5A and B), indicative of reduced β-cell proliferation. Analysis of BrdU incorporation into nuclei of glucagon- or somatostatin- immunoreactive cells in pancreases from P9 WT and *Gpr56* KO mice indicated that there was a trend towards enhanced α-cell proliferation (Fig. 5C and D) and a significant increase in δ-cell proliferation (Fig. 5E and F). Overall, *Gpr56* deletion was not associated with a significant alteration in islet size (WT: 3,286±821μm^2^; KO: 2,177±926μm^2^, *n* = 4 mice per genotype, *p* > 0.2).

## Discussion

There is a wealth of evidence supporting key roles for aGPCRs in appropriate organ development [10–17], but very little is known about their importance for islet cell differentiation. In this study, our analysis of our own and publicly available data has revealed that transcripts encoding several aGPCRs and their ligands are expressed by both developing mouse and human pancreas. We found that while there is conserved expression of some aGPCRs in mouse and human pancreas, there are important differences in their distribution and expression among the different pancreatic cell types. Thus, in both species, CELSR2 and GPR56 were the most abundant aGPCR mRNAs expressed by the endocrine progenitors, with GPR56 being the highest. A previous report has indicated that Celsr2 and Celsr3 are required for differentiation of mouse pancreatic endocrine progenitors into β-cells [54], and our observation in human fetal pancreas of increases in CELSR2 and CELSR3 mRNA expression from 7 to 10 PCW, a period of endocrine progenitor differentiation [42], suggests that these aGPCRs may also be involved in β-cell specification from endocrine progenitors in human pancreas. We have previously reported that GPR56 is the most abundant GPCR in isolated mouse and adult human islets [24, 26] and that Gpr56 activation in islets potentiated glucose stimulated insulin secretion [27].

We also measured the expression patterns of all aGPCR mRNAs at multiple stages of human pancreas development and found that expression of mRNAs encoding LPHN1, LPHN2, GPR124 and GPR125 were consistently elevated throughout gestation. These receptors have been associated with vascular morphogenesis, cell migration, pluripotency and neural development in other tissues [52, 55–57], consistent with their involvement in human endocrine pancreas development. Most aGPCRs are orphan receptors with no known ligands, but the endogenous ligands of deorphanised aGPCRs are mainly components of the extracellular matrix. We found that mRNAs encoding these extracellular matrix components are present in human and mouse pancreatic mesenchymal, endothelial and Schwann cells, all of which are localised in the developing islet microenvironment [31], and so may influence islet cell differentiation via cell-cell contact or paracrine signalling.

We here demonstrate that GPR56 mRNA was the most highly expressed aGPCR in mouse and human fetal pancreas endocrine progenitor cells and that its expression was upregulated during human β-cell development between 10 and 17 PCW, suggesting a role in β-cell development. GPR56 was ubiquitously expressed by pancreatic cells, but its expression became progressively restricted to β-cells during mouse and human pancreas development. We have previously shown that the Gpr56 ligand collagen III is present in peri-islet and peri-vascular basement membrane regions within mature mouse islets where it exerts beneficial effects on islet function through activation of GPR56 [27], consistent with effects on β-cell development. To evaluate the requirement of Gpr56 for appropriate islet endocrine cell distribution in postnatal mice, we used a mouse model in which *Gpr*56 is globally deleted. We selected a global knockout model because in an insulin promoter-driven β-cell-specific *Gpr*56 knockout model Gpr56 deletion would only occur after β-cell specification, so it would not allow us to investigate a role for Gpr56 in β-cell development. We found that deletion of Gpr56 led to a reduction in β-cell number with decreased β-cell proliferation and a concomitant increase in the number of α-cells and δ-cells. Together these data suggest that Gpr56 activation, perhaps by collagen III, is necessary for normal development of β-cells.

In summary, we here present a comprehensive mRNA expression profile of aGPCRs during mouse and human pancreas development, and we have identified differential expression patterns among the different pancreatic cells. In particular, the expression pattern of mRNAs encoding GPR56 and its ligand collagen III implicate them in β-cell differentiation during normal development. Current efforts to generate functional β-cells from pluripotent stem cells are progressing [58] but are not yet optimal. We suggest that differentiation protocols may be improved by consideration of the potential roles of aGPCRs, particularly GPR56, in β-cell differentiation.

## Electronic supplementary material

Below is the link to the electronic supplementary material.

Supplementary Material 1

## Data Availability

The human single cell RNA seq and spatial transcriptomics data are available from GEO (GSE197064 and GSE197317) as reported previously [31].
