# Supplementary material for: Spatiotemporal profiling of adhesion G protein-coupled receptors in developing mouse and human pancreas reveals a role for GPR56 in islet development
Source: Cell Mol Life Sci. 2025 Mar 26;82(1):129. doi: 10.1007/s00018-025-05659-z (PMC11947406; doi:10.1007/s00018-025-05659-z)
Supplement: Supplementary file 1 — Supplementary Material 1 [file 18_2025_5659_MOESM1_ESM.docx]

SUPPLEMENTARY FIGURES

| 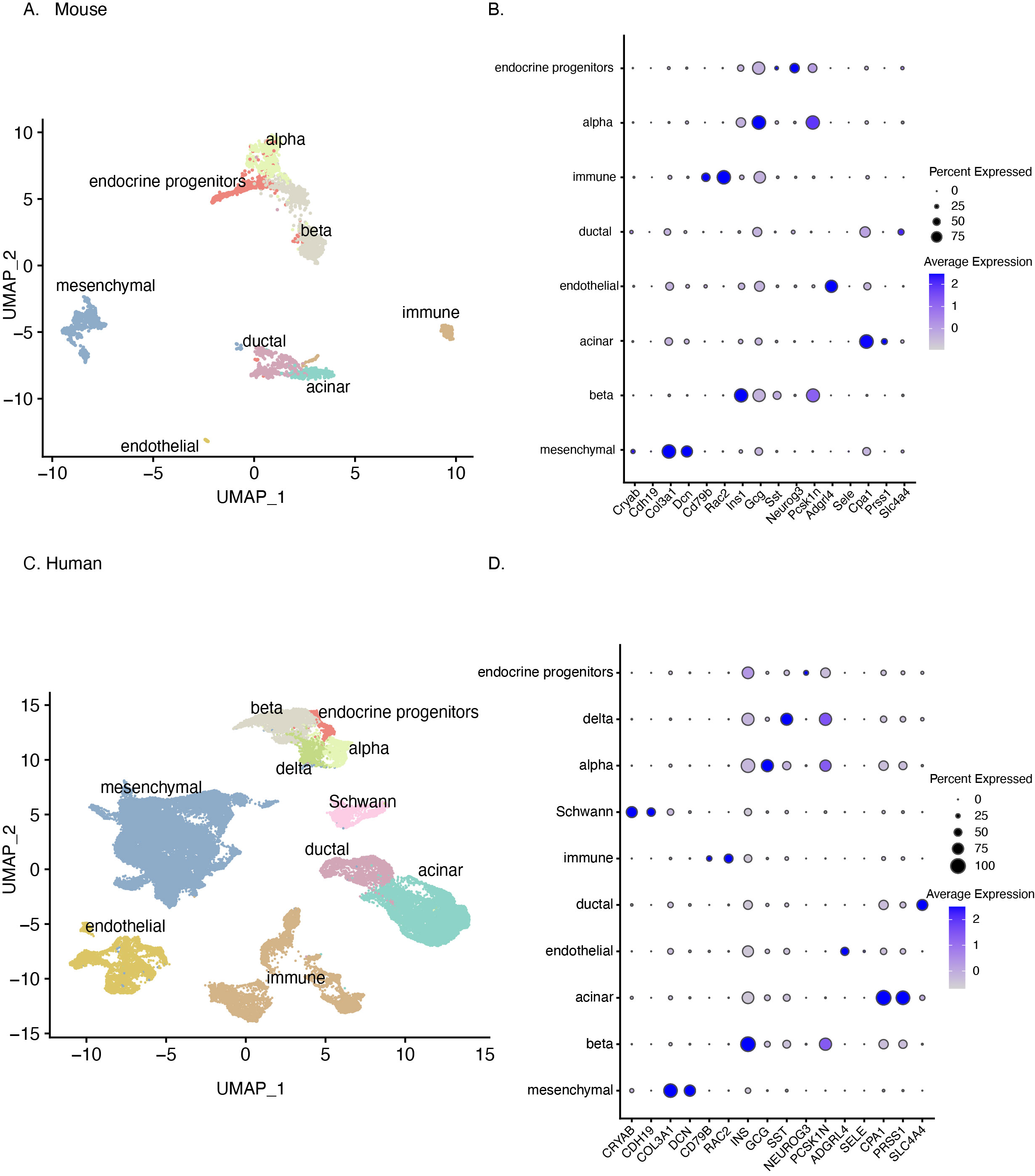 |
| --- |

**Suppl. Fig. 1.** **Different cell types in mouse and human pancreases from the single cell RNA sequencing data.**

A, C) UMAP embedding showing the various cell types in mouse and human pancreases. B, D) Dotplots showing marker gene expression and distribution in mouse and human pancreases.

| 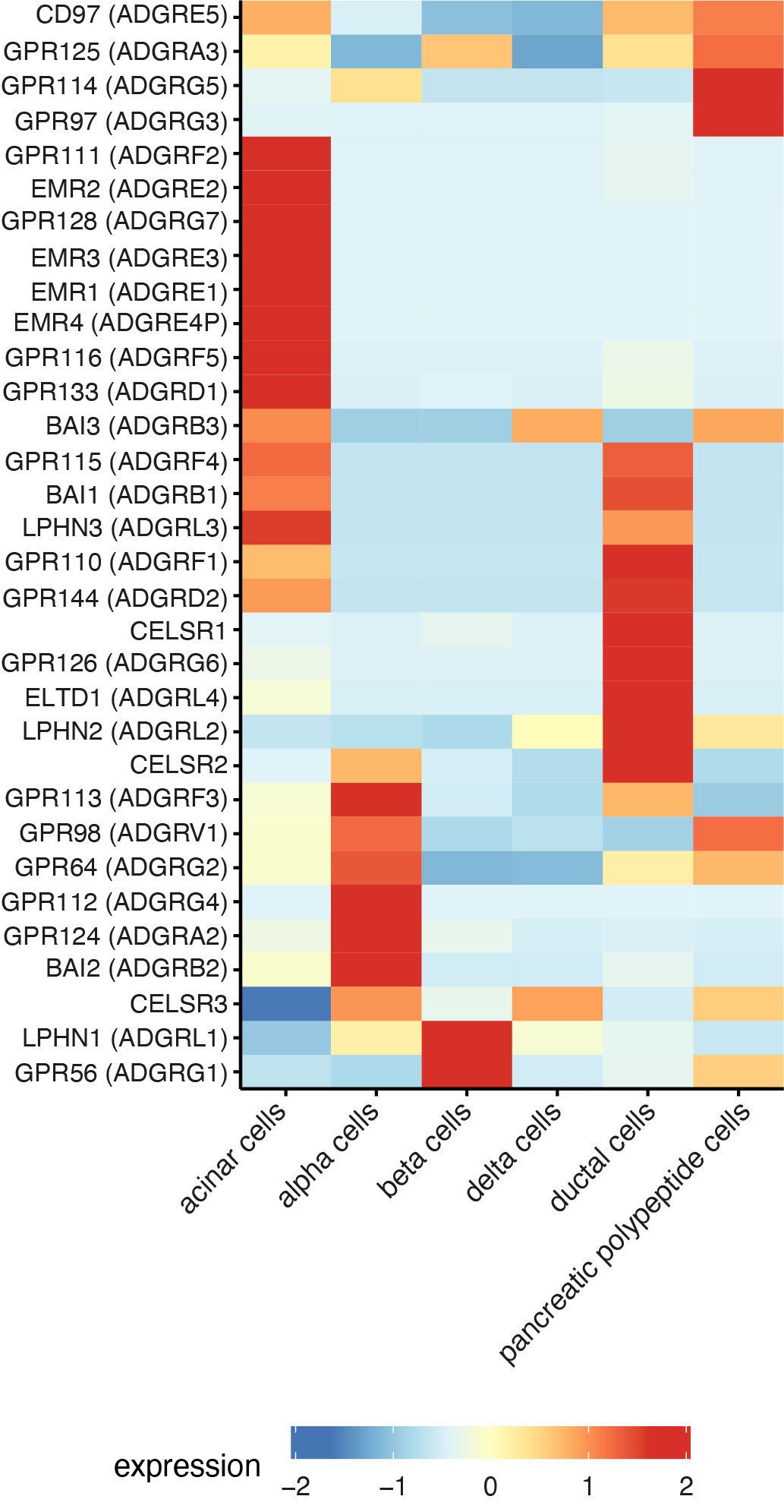 |
| --- |

**Suppl. Fig. 2.** **Expression of aGPCRs in different cell types of the adult pancreas.**

A heatmap showing expression of aGPCRs in different cell types of adult human pancreas. Data were retrieved from published scRNAseq analysis of adult pancreases from 15 donors [35].

| 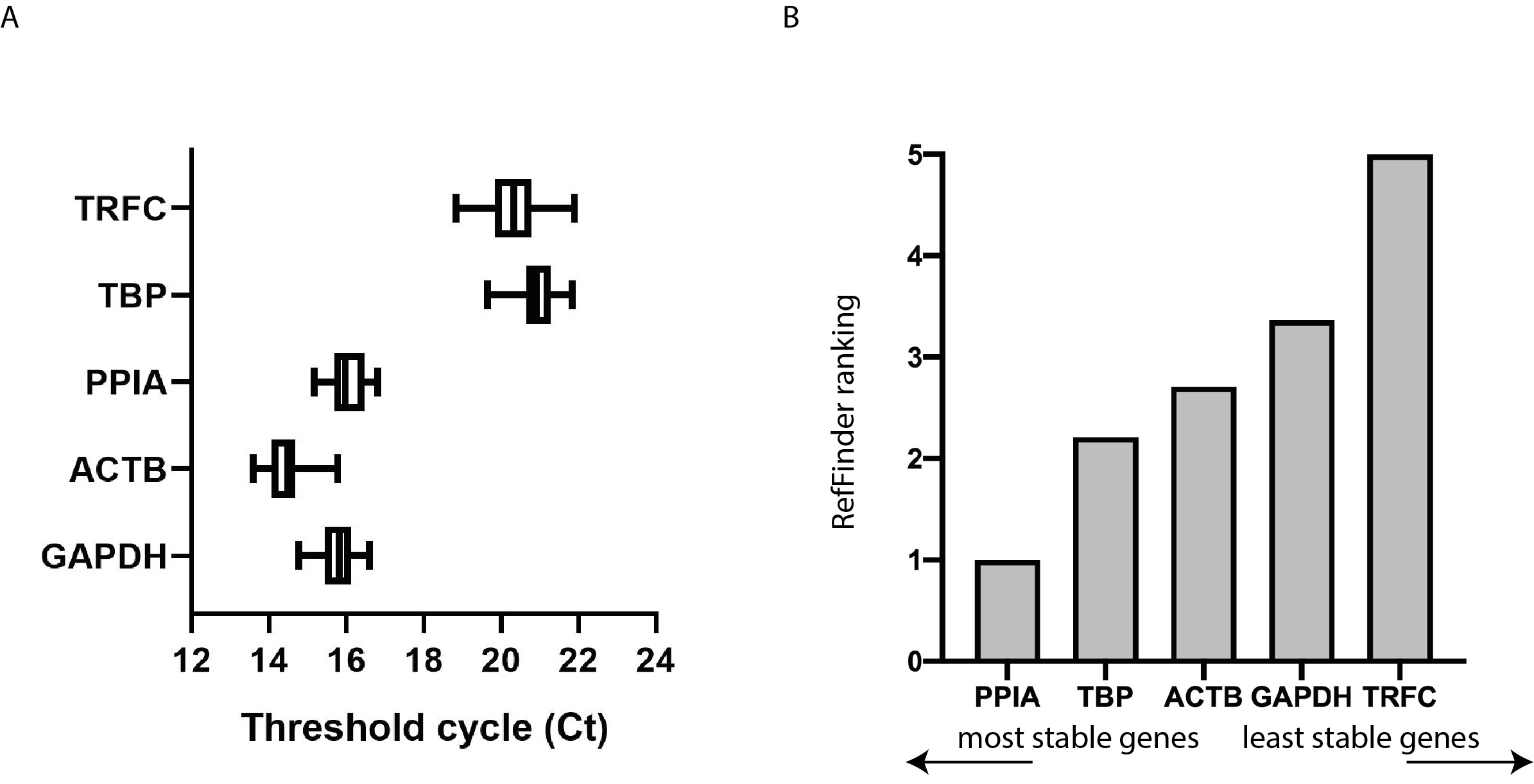 |
| --- |

**Suppl. Fig. 3.**

**Selection of reference genes for qPCR profiling of adhesion GPCR mRNA expression.**

A) qPCR threshold cycles for five reference genes in human fetal pancreas.

B) RefFinder ranking of the most stable and least stable reference genes over the human fetal pancreas developmental timepoints.

| 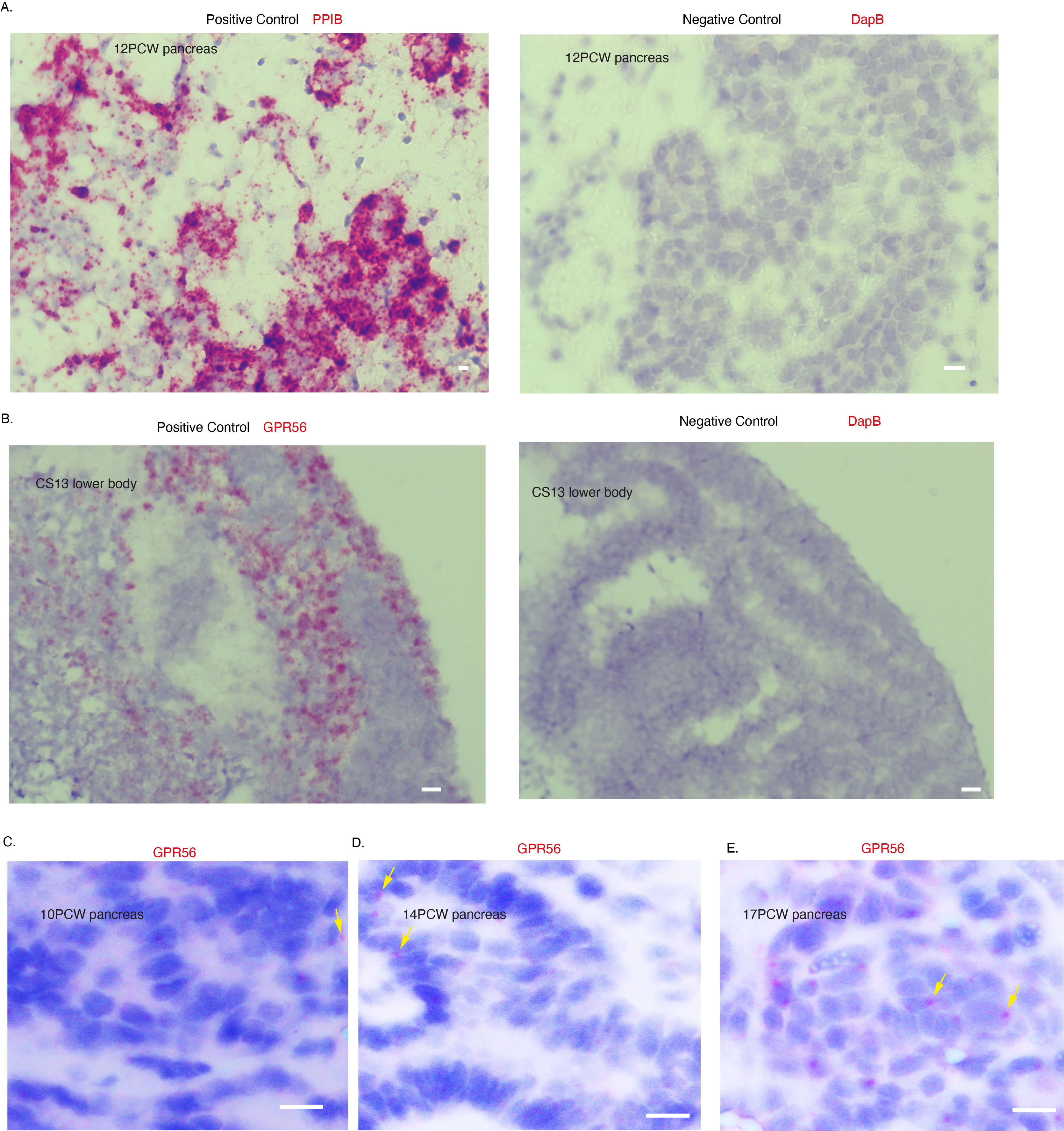 |
| --- |

**Suppl. Fig. 4. RNAscope analysis of GPR56 mRNA expression in developing human pancreas.**

1. Expression of positive control RNA (PPIB; red) and negative control staining (DapB) in human pancreas at 12PCW. Scale bar: 10μm.
2. Expression of GPR56 mRNA (red) and negative control staining (DapB) in a Carnegie stage (CS) 13 embryo (lower body). Scale bar: 10μm.

C-E) Expression of GPR56 mRNA (red) in human pancreas sections at 10, 14 and 17 PCW. Yellow arrows indicate single GPR56 mRNA molecules. Blue DAPI staining indicates nuclei. Scale bar: 10μm.
